# Validation of a survey methodology for gastroesophageal reflux disease in China

**DOI:** 10.1186/1471-230X-8-37

**Published:** 2008-08-21

**Authors:** Yang Cao, Xiaoyan Yan, Xiu-Qiang Ma, Rui Wang, Saga Johansson, Mari-Ann Wallander, Jia He

**Affiliations:** 1 Department of Health Statistics, Second Military Medical University, Shanghai, PR China; 2 AstraZeneca R&D, Mölndal, Sweden; 3 Institute of Medicine, Sahlgrenska Academy, Gothenburg University, Sweden; 4 Department of Public Health and Caring Science, Uppsala University, Sweden

## Abstract

**Background:**

Gastroesophageal reflux disease (GERD) causes a wide range of clinical symptoms and potentially serious complications, but epidemiological data about GERD in China are limited. The aim of this pilot study was to develop and validate a methodology for the epidemiological study of GERD in China.

**Methods:**

Regionally stratified, randomized samples of Shanghai residents (n = 919) completed Mandarin translations of the Reflux Disease Questionnaire (RDQ), GERD Impact Scale, Quality of Life in Reflux and Dyspepsia (QOLRAD) questionnaire and 36-item Short Form Health Survey (SF-36). Reliability and construct validity were tested by appropriate statistical analyses.

**Results:**

The response rate was 86%. The test-retest reliability coefficients for the RDQ, GERD Impact Scale, QOLRAD and SF-36 were 0.80, 0.71, 0.93 and 0.96, respectively, and Cronbach's alpha coefficients were 0.86, 0.80, 0.98 and 0.90, respectively. Dimension scores were highly correlated with the total scores for the QOLRAD and SF-36, and factor analysis showed credible construct validity for the RDQ, GERD Impact Scale and SF-36. The RDQ GERD score was significantly negatively correlated with QOLRAD dimensions of food and drink problems and social functioning, and was significantly negatively correlated with all dimensions of the SF-36. All eight of the SF-36 dimensions were significantly correlated with the QOLRAD total score.

**Conclusion:**

This study developed and tested a successful survey methodology for the investigation of GERD in China. The questionnaires used demonstrated credible reliability and construct validity, supporting their use in larger epidemiological surveys of GERD in China.

## Background

Gastroesophageal reflux disease (GERD) is a common disorder caused by backflow of stomach contents into the esophagus. As it can cause a wide range of clinical symptoms and potentially serious complications, the epidemiology of GERD has been a subject of much interest in recent years. GERD is frequently diagnosed on the basis of symptoms alone, with the criterion for diagnosis in clinical practice being when reflux symptoms become troublesome to the patient [1]. However, for epidemiological studies, a simple symptom threshold is required to identify those who have GERD. In many studies, this threshold is defined as at least weekly reflux symptoms [2]. GERD is common in the West, with a prevalence of about 10–20%, but the prevalence in Asia is generally lower at approximately 5% [2]. The prevalence of GERD is, however, thought to be increasing [3], with trends in Asia attracting particular interest [4]. There have been few high quality, population-based epidemiological surveys of GERD in Asia, particularly in China [5]. A number of methodological challenges associated with studying the epidemiology of GERD in this region may have contributed to this paucity.

To identify reflux symptoms accurately, validated patient-completed questionnaires are needed, as clinicians tend to underestimate the presence and severity of reflux symptoms reported by patients [6]. In particular, validated symptom descriptors (e.g. 'burning behind the breastbone') are necessary because terms such as 'heartburn' are known to be poorly understood by patients [7]; this is of particular relevance to Chinese populations, because there is no word for 'heartburn' in Mandarin Chinese beyond specialist medical circles, and a survey in the USA revealed that only 13.2% of East Asian patients understood the term [7].

Within the Chinese population, language and cultural differences can lead to different communities perceiving and expressing their symptoms differently. In China, Mandarin is the official language, but about half the population does not speak it, particularly those living in rural areas and older people [8]. There are thousands of local dialects, many of which are mutually unintelligible when spoken. All use the same writing system, and overall literacy rates in China are high, but literacy among older people, women and those living in rural areas is relatively low; in the 2003 census, over 9.6% of women and 2.1% of men were illiterate or semi-literate [9].

Population surveys can be difficult to implement in China. Telephone surveys may introduce population bias in favour of the more wealthy urban Chinese population who are more likely to have telephones. The utility of postal surveys is limited by the ability of the respondent to understand the terms used [10] which, for questionnaires developed in the West, may be further compounded by cultural conceptual differences. Response rates to telephone or postal questionnaires may be low, potentially introducing responder bias [11,12]. For these reasons, previous population surveys of GERD in China have administered questionnaires using a face-to-face interview technique, in which subjects completed the questionnaire while being assisted by trained interviewers [10,13,14]. This technique has achieved high response rates and has enabled terms and definitions to be clarified appropriately for individual respondents.

In order to investigate the prevalence and impact of GERD in China and facilitate comparisons with other countries, linguistic and psychometric validation of internationally recognized disease-specific and generic patient-reported outcomes instruments is required. The aim of this pilot study was to develop and validate a methodology for the epidemiological study of GERD in China. The feasibility, validity and reliability of several well-designed questionnaires were tested in a Chinese environment using randomized, stratified, multi-stage cluster sampling, a statistical sampling technique adopted by the World Health Organization (WHO) [15] that is particularly well suited to the residential and social administration system in China.

## Methods

### Setting

**Figure 1:**
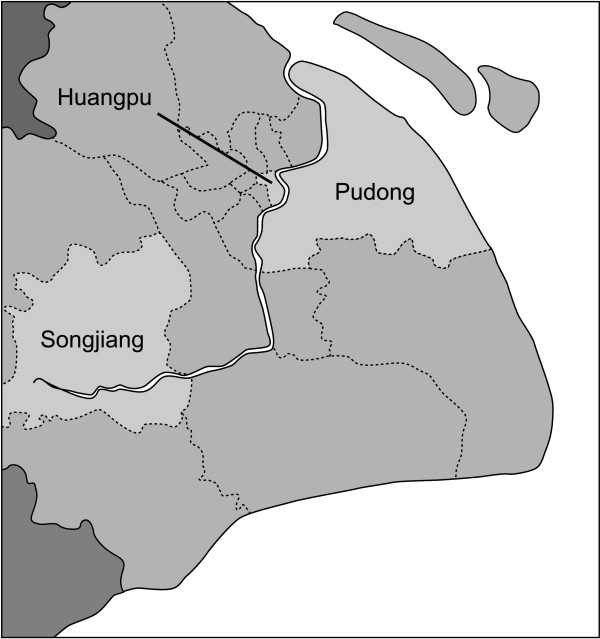
The survey sites in Shanghai.

Shanghai, on the east coast of China, is China's largest city. It is divided into 18 districts and one county, each of which is classified as urban, suburban, or rural (Figure 1). Each district includes numerous blocks, which include multiple residential areas, and the county covers several towns that govern a number of villages. Broadly speaking, people who live in an urban area have a city lifestyle, while people who live in a rural region lead a farming or country peasant way of life. The suburban lifestyle is intermediate between these two.

### Sampling

**Figure 2:**
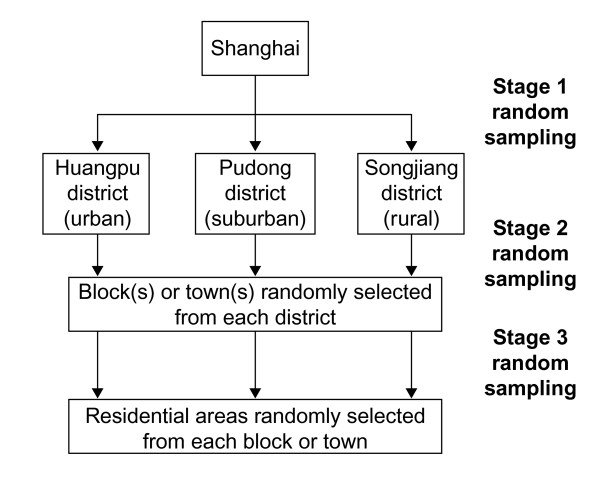
Stratified, multi-stage randomized cluster sampling of urban, suburban and rural districts in Shanghai.

A randomized, stratified, multi-stage cluster sampling methodology was used to select a representative sample of the general population in Shanghai. Huangpu was randomly selected from the nine urban districts, Pudong from the four suburban districts, and Songjiang from the five rural districts and one county of Shanghai. Blocks were randomly selected from districts and residential areas from blocks so that, finally, four residential areas in the urban district, three in the suburban district and two in the rural district were randomly selected (see Figures 1 and 2). The Residential Committee of each residential area supplied detailed household rosters of all adults, and subjects for this study were randomly sampled from these lists.

Pudong District consists of 26 towns and blocks, and is the biggest district in Shanghai. The residents in this district are widely dispersed and not all the information for each resident could be obtained. As information for all families in Pudong was available, families were randomly sampled from the selected residential areas and the family member with a birthday closest to the investigation date was selected.

According to the statistical formula n = t^2^pq/d^2 ^(where n, t, p, q and d are sample size, t value, positive rate, negative rate and acceptable error, respectively), assuming a GERD prevalence of 10%, and setting significance at P = 0.05 and acceptable error at 2%, the calculated sample size was 864 [16]. According to the 1 in 10 000 sampling proportion principle and the population size of Shanghai, the target sample size was 1300 respondents. Combining these two figures, a target sample size of 1000 valid respondents was deemed appropriate. Allowing for a 20% non-response rate, the final intended sample size was set at 1200, including 400 subjects from each district.

Residents under 18 years of age, or residents who were illiterate, had severe visual, hearing or learning disabilities, or major psychiatric illness, were excluded from the survey. Respondents who were not at home after three attempts to administer the questionnaire were considered to be missing.

### Administration of questionnaires

Local residential committee staff informed residents of the survey and secured their support and understanding. The informed consent of respondents was obtained, and each respondent was free to discontinue participation in the study at any time. The study was approved by the Second Military Medical University Ethics Committee.

During the fieldwork period from November 2005 to January 2006, respondents completed questionnaires in their own homes or in local residential committee offices. Questionnaires were self-administered, with trained and supervised facilitators on hand to explain any questions that were unclear. The facilitators were social workers at the site, who were trained by supervisors who were professionals and graduate students from the Department of Health Statistics (DoHS), who received training from an epidemiology survey expert from the DoHS and a gastrointestinal specialist from Shanghai Hospital. Quality auditing was performed to ensure all questionnaires were completed properly. A valid questionnaire was one that had been audited and signed by a supervisor.

### Questionnaires

Each respondent completed five questionnaires in Mandarin (see additional file 1: GERD questionnaire in English and Mandarin Chinese): a general information questionnaire and translations of four concise, well-validated, internationally recognized and frequently cited disease-specific and generic health questionnaires, chosen to facilitate comparison with other studies and minimize the length of the overall survey: the Reflux Disease Questionnaire (RDQ), the GERD Impact Scale, the Quality of Life in Reflux and Dyspepsia (QOLRAD) questionnaire, and the 36-Item Short-Form Health Survey (SF-36). The general information questionnaire collected information on age, gender, education, income and other general demographic variables.

The RDQ is a 12-item self-report questionnaire measuring the frequency and severity of upper gastrointestinal symptoms (heartburn, regurgitation and epigastric pain) over the previous week. Symptom frequency and severity are scored on a 6-point Likert scale (0–5, where 5 is the most severe/frequent). A GERD dimension score can be obtained by combining the heartburn and regurgitation scores [17]. Subjects reporting heartburn and/or regurgitation of any frequency during the 1-week recall period of the questionnaire were defined as having GERD. The RDQ was validated for use in clinical trials in two large studies [18,19], and was also recently validated for use as a diagnostic tool in the DIAMOND study (Diagnostic Tool for the Management of Patients with Reflux Disease) [20]. A Chinese version of the RDQ was tested in 10 hospitals in mainland China, and was found to identify accurately the presence of symptoms suggestive of GERD experienced over the previous month [21].

The GERD Impact Scale questionnaire is an eight-item self-report questionnaire designed to aid patient-physician communication in primary care. It assesses the frequency of gastroesophageal reflux symptoms over the past 2 weeks and their impact on everyday activities such as sleep, work, meals and social occasions, and the use of additional medication (other than that prescribed). Four response options for frequency are provided (1–4) where 1 is 'all of the time' and 4 is 'none of the time'. This newly developed tool has demonstrated good psychometric properties [22].

The GERD-specific version of the QOLRAD questionnaire is a 25-item disease-specific quality-of-life instrument measuring the impact of upper gastrointestinal symptoms over the previous week on five dimensions: emotional well-being, sleep, vitality, eating/drinking, and physical/social functioning [23]. The frequencies of effects are reported using a 7-point Likert scale, with low scores indicating frequent impairment. Its reliability and validity have been extensively documented in studies of patients with upper gastrointestinal symptoms [23-25].

The SF-36 is a generic questionnaire assessing health status and well-being over the past 4 weeks. It contains 36 items clustered in eight dimensions: physical functioning, role-physical, bodily pain, general health, vitality, social functioning, role-emotional, and mental health, plus one item assessing change in health status over the previous year [26]. Item scores for each dimension are coded, summed and transformed to a scale from 0 (worst possible health state) to 100 (best possible health state). Its reliability and validity are widely documented across a range of language versions [27,28].

### Translation and cognitive debriefing

Apart from the SF-36, where validated Mandarin translations already exist [29], questionnaires were translated and tested in the Department of Medicine, Faculty of Medicine, at the University of Hong Kong. Literal translation of Hong Kong Chinese into mainland Chinese (Mandarin) was undertaken by investigators and a panel of mainland gastroenterologists so that questionnaires were more interpretable by people from mainland China. This process was followed by cognitive debriefing, where five literate volunteers from mainland China who had a diagnosis of GERD (heartburn and/or acid regurgitation over the past year) completed the translated questionnaires and were interviewed to assess their understanding and interpretation. The overall relevance and clarity of the questionnaire were assessed using defined responses (very low; low; moderate; high; very high) and subjects were asked to specify any items that they regarded as irrelevant or unclear. Subjects considered the questions to be relevant and clear (grading: moderate to very high). No additional revisions were required.

### Statistical analysis

#### Data management

Questionnaire responses were coded and double-entered by two independent professional data-entry staff from the DoHS. EpiData software [30] was used to check for consistency between the two sets of data entries to ensure data quality. For the RDQ, QOLRAD, and SF-36, where at least 50% of items in a dimension were completed, the mean value of the completed items was used to impute the missing values. Where more than 50% of items were missing, the dimension score was excluded from the analysis [31-33]. For the GERD Impact Scale, if an item score was missing, imputation was not performed and the score was excluded from the analysis.

SAS 9.1.3 (SAS, Shanghai, China) and SPSS 10.0 software (SPSS Inc., Shanghai, China) were used to complete data analyses. All hypothesis tests used two-side tests and set alpha at 0.05. A two-tailed *P*-value of 0.05 or less was considered to indicate statistical significance. Different groups of subjects were compared by ANOVA for normally distributed continuous data, Fisher's exact test for categorical variables and the Cochran-Mantel-Haenszel test for ranked variables.

#### Reliability

Internal consistency was evaluated using Cronbach's alpha coefficient to determine the extent to which items within each questionnaire were interrelated [34]. Cronbach's alpha coefficients for each questionnaire were calculated by correlating all individual item scores with dimension scores and/or the overall score. An alpha coefficient above 0.70 suggests good internal consistency and reliability.

Test-retest reliability is a measure of the stability of the instrument under different conditions with the same respondent; in this study, it was assessed by retesting 10% of respondents (n = 40 from each region) 2–7 days after the baseline test. Cohen's kappa coefficient and the intraclass correlation coefficient (ICC) were used to analyze the test-retest reliability of the survey instruments. Cohen's kappa coefficient was used in the analysis of categorical and ranked measurements, while ICC was used to analyze quantitative measurements. A test-retest coefficient above 0.70 was considered acceptable [35].

#### Construct validity

Construct validity evaluates whether an instrument actually measures the phenomena that it theoretically predicts; correlation and factor analysis were used to evaluate construct validity in this study. Factor analysis using principal component analysis and quartimax rotation explored whether the factor structure of each questionnaire was supported. Factor loadings larger than 0.50 within one dimension were considered to support the factor construct provided the factor loadings were low across the other dimensions, with cumulative rates used to show the contributions of combinations of principal components [36]. Correlation analysis tested the construct validity of questionnaires containing multiple dimensions (i.e. RDQ, QOLRAD and SF-36). The analysis measured the strength of association between dimension scores and the total score for QOLRAD and SF-36 questionnaires, and between item scores and dimension scores for the RDQ. A strong correlation coefficient was considered to be over 0.6, a moderate correlation, 0.3–0.6, and a weak correlation below 0.3 [37].

Convergent validity analyzes whether the postulated dimension of an instrument correlates appreciably with all other dimensions from other instruments that should theoretically be related to it. Convergent validity was investigated in this study by correlating the GERD dimension from the RDQ with SF-36 and QOLRAD dimensions, and SF-36 dimensions with QOLRAD total score. A decrease in health-related quality of life was expected for respondents with GERD symptoms.

## Results

### Response rate

Of the 1200 randomly pre-selected subjects, 1034 agreed to be interviewed (a response rate of 86%). In the Pudong District, a total of 112 respondents' questionnaires were withdrawn from the statistical analysis due to one facilitator's failure to adhere to the study protocol. A further three questionnaires from the Huangpu District were excluded due to incompleteness. Therefore, a total of 919 questionnaires (359 from the urban region, 224 from the suburban region, and 336 from the rural region) were included in the analysis after quality auditing. The mean response rates for items in each questionnaire are provided in Table 1.

**Table 1:** Mean item response rates by questionnaire and by region.

	**Mean item response rates**			
	
Region	RDQ	GERD Impact Scale	QOLRAD	SF-36
Urban (n = 359)	98.35–98.62%	98.07–98.35%	96.97–100%	98.07–99.45%
Suburban (n = 224)	100–100%	100–100%	100–100%	98.21–100%
Rural (n = 336)	100–100%	100–100%	94.11–100%	97.92–99.70%
All regions (n = 919)	99.35–99.46%	99.24–99.35%	98.25–100%	98.59–99.67%

GERD: gastroesophageal reflux disease; QOLRAD: Quality of Life in Reflux and Dyspepsia questionnaire; RDQ: Reflux Disease Questionnaire; SF-36: 36-item Short-Form Health Survey.

Of 120 subjects randomly selected for retest, 113 agreed to be re-interviewed (a 94% response rate). Fourteen questionnaires were rejected because they were not completed in line with the study protocol, leaving 99 questionnaires for inclusion in the retest analysis.

### Respondents

The respondents' average age was 47 years (ranging from 18 to 77 years); 55% were female and the majority of respondents (85%) were married. Most respondents did not smoke (74%) or drink alcohol (83%). The average BMI was 22.6 kg/m^2^, with a range of 14.4–36.5 kg/m^2^. Level and years of education, current job type and income level all varied significantly between the three regions (p < 0.0001). Education levels and family income were greatest for the urban region and lowest for the rural region (Table 2), reflecting the socioeconomic divide that exists between urban and rural China. Forty percent of urban respondents were professionals or technicians, while 73% of rural respondents and 44% of suburban respondents were agricultural or fishery workers.

**Table 2:** Demographics and baseline characteristics of respondents by region.

	Mean ± SD or number of subjects (%)			
**Variables**	Urban (n = 359*)	Suburban (n = 224*)	Rural (n = 336*)	Total (n = 919*)

Age (years)	45.7 ± 14.5	45.2 ± 13.1	49.0 ± 12.2	46.7 ± 13.4
Weight (kg)	63.0 ± 11.0	61.2 ± 10.1	59.3 ± 8.2	61.2 ± 10.0
Height (cm)	167.3 ± 8.2	162.8 ± 7.7	162.0 ± 6.8	164.3 ± 8.0
**Body mass index (kg/m**^2^)	22.4 ± 3.0	23.1 ± 3.3	22.6 ± 2.7	22.6 ± 3.0
**Sex**				
Female	193 (53.8)	139 (62.1)	177 (52.7)	509 (55.4)
Male	166 (46.2)	85 (38.0)	159 (47.3)	410 (44.6)
**Marital status**				
Married	266 (75.1)	197 (88.7)	306 (94.2)	796 (85.4)
Unmarried	88 (24.9)	25 (11.3)	19 (5.9)	132 (14.7)
**Maximum education level**				
Primary school/uneducated	4 (1.1)	61 (27.2)	189 (56.8)	254 (27.7)
Secondary/high school	232 (64.6)	138 (61.6)	139 (41.7)	509 (55.6)
College graduate or beyond	123 (34.3)	25 (11.2)	5 (1.5)	153 (16.7)
Years of school education	12.3 ± 2.5	8.9 ± 3.7	6.2 ± 3.6	9.3 ± 4.2
**Current job**				
Government employee	20 (5.6)	1 (0.5)	1 (0.3)	22 (2.4)
Professional or technician	144 (40.3)	18 (8.0)	12 (3.6)	174 (19.02)
Blue-collar worker	70 (19.6)	40 (17.9)	51 (15.3)	161 (17.6)
Agricultural or fisheries worker	1 (0.3)	98 (43.8)	243 (72.8)	342 (37.4)
Student in school	30 (8.4)	7 (3.1)	4 (1.2)	41 (4.5)
Others	92 (25.7)	60 (26.8)	23 (6.9)	175 (19.1)
**Total family income per month**^†^				
less than 1999 Yuan	178 (50.9)	124 (57.1)	232 (69.2)	534 (59.2)
2000–4999 Yuan	157 (44.9)	81 (37.3)	98 (29.3)	336 (37.3)
5000–9999 Yuan	13 (3.7)	12 (5.4)	4 (1.2)	29 (3.2)
10000 Yuan or above	2 (0.6)	0 (0)	1 (0.3)	3 (0.3)
**Smoking**				
No	282 (78.6)	163 (73.4)	225 (68.2)	670 (73.6)
Yes	77 (21.5)	59 (26.6)	105 (31.8)	241 (26.5)
Years of smoking	22.5 ± 11.6	20.6 ± 12.6	19.63 ± 11.7	20.8 ± 11.9
**Alcohol intake**				
No	308 (85.8)	193 (87.3)	251 (76.1)	752 (82.6)
Yes	51 (14.2)	28 (12.7)	79 (23.4)	158 (17.4)
Years of alcohol intake	18.7 ± 11.1	21.06 ± 11.9	18.26 ± 10.9	18.69 ± 11.1

*Totals may not tally exactly where individual subjects have refused to answer a specific question.

^†^2000 Yuan is equivalent to approximately 260 USD, at an approximate exchange rate of 7.7 Yuan = 1 USD (April 2007).

### Reliability

In the test-retest analysis, Cohen's kappa coefficients ranged from 0.66 to 1.00 for RDQ dimensions, 0.49 to 1.00 for GERD Impact Scale items, and 0.79 to 1.00 for QOLRAD dimensions. The test-retest ICC ranged from 0.69 to 0.97 for seven dimensions of the SF-36 questionnaire, while one (role-emotional) was close to zero (0.01). Internal consistency (indicated by Cronbach's alpha coefficient) ranged from 0.65 to 0.97 for QOLRAD dimensions. For SF-36, seven dimensions ranged from 0.69 to 0.95, while one (social functioning) was 0.31. The test-retest reliability coefficient and total Cronbach's alpha coefficient for each questionnaire are shown in Table 3. All coefficients were ≥ 0.7, demonstrating good reliability and internal consistency for each questionnaire.

**Table 3:** Reliability of questionnaires.

**Questionnaire**	**Number of items**	Test-retest reliability coefficient*	**Cronbach's alpha coefficient**
RDQ	12	0.80	0.86
GERD Impact Scale	8	0.71	0.80
QOLRAD	25	0.93	0.98
SF-36	36	0.96	0.90

*Cohen's kappa coefficient for the RDQ, GERD Impact Scale and QOLRAD; intraclass correlation coefficient for SF-36. GERD: gastroesophageal reflux disease; RDQ: Reflux Disease Questionnaire; QOLRAD: Quality of Life in Reflux and Dyspepsia questionnaire; SF-36: 36-Item Short-Form Health Survey.

### Construct validity

Each dimension score was highly correlated with the total score for both QOLRAD and SF-36 (p < 0.001), indicating good construct validity. For QOLRAD, Spearman correlation coefficients ranged from 0.77 for physical/social functioning to 0.91 for food and drink problems and for vitality, among respondents reporting symptoms of heartburn and/or regurgitation via the RDQ. For SF-36, Spearman correlation coefficients ranged from 0.53 for social functioning to 0.77 for general health, for the study population as a whole. The RDQ also demonstrated good construct validity (Table 4), with each dimension correlating most strongly with the individual items comprising it (Spearman correlation coefficients 0.62–0.94). Regurgitation items correlated strongly with the GERD dimension as expected, but the weaker correlation with heartburn items may have been due to the low prevalence of heartburn in the Shanghai population.

**Table 4:** Spearman correlation coefficient between RDQ item score and RDQ dimension score.

**RDQ item**	**Heartburn dimension**	**Regurgitation dimension**	**GERD dimension**	**Epigastric pain dimension**
Burning behind breastbone – severity	**0.62**	0.34	0.38	0.32
Burning behind breastbone – frequency	**0.62**	0.34	0.38	0.32
Pain behind breastbone – severity	**0.93**	0.32	0.56	0.59
Pain behind breastbone – frequency	**0.93**	0.32	0.56	0.59
Acid taste – severity	0.32	**0.91**	**0.84**	0.44
Acid taste – frequency	0.32	**0.91**	**0.84**	0.43
Movement of materials – severity	0.42	**0.72**	**0.67**	0.39
Movement of materials – frequency	0.43	**0.72**	**0.67**	0.39
Upper stomach burning – severity	0.54	0.41	0.44	**0.68**
Upper stomach burning – frequency	0.54	0.41	0.44	**0.68**
Upper stomach pain – severity	0.58	0.45	0.56	**0.94**
Upper stomach pain – frequency	0.58	0.45	0.56	**0.94**

Numbers in bold indicate correlation coefficient ≥ 0.6.

GERD: gastroesophageal reflux disease; RDQ: Reflux Disease Questionnaire

Factor analysis was used to explore whether the predicted factor structure of the questionnaire was supported. Credible construct validity was demonstrated for the RDQ, GERD Impact Scale and SF-36 questionnaires. All RDQ items correlated as expected in the factor analysis apart from the frequency and severity of 'pain behind breastbone', which correlated more strongly with the epigastric pain dimension than the heartburn dimension (Table 5). The cumulative rate of the three factors was 72.1%. All GERD Impact Scale items correlated with factors as expected (Table 6). The cumulative rate of the four factors was 78.0%.

**Table 5:** Factor analysis matrix of RDQ items in three factors (heartburn, regurgitation and epigastric pain).

**Symptom**	**Heartburn**	**Regurgitation**	**Epigastric pain**
**Heartburn**			
Burning behind breastbone – severity	**0.84**	0.22	0.31
Burning behind breastbone – frequency	**0.84**	0.11	0.37
Pain behind breastbone – severity	0.25	0.05	**0.80**
Pain behind breastbone – frequency	0.10	0.04	**0.84**
**Regurgitation**			
Acid taste – severity	-0.09	**0.89**	0.16
Acid taste – frequency	-0.07	**0.84**	0.15
Movement of materials – severity	0.33	**0.72**	0.17
Movement of materials – frequency	0.36	**0.72**	0.17
**Epigastric pain**			
Upper stomach burning – severity	0.31	0.12	**0.59**
Upper stomach burning – frequency	0.36	0.01	**0.63**
Upper stomach pain – severity	-0.18	0.27	**0.83**
Upper stomach pain – frequency	-0.15	0.14	**0.88**

Numbers in bold indicate correlation coefficient ≥ 0.5.

RDQ: Reflux Disease Questionnaire.

**Table 6:** Factor analysis matrix of GERD Impact Scale items in four factors.

**Symptom (frequency)**	**Regurgitation and effect on eat/sleep/work**	**Heartburn and effect on sleep**	**Additional medication**	**Sore throat/hoarseness**
Pain behind breastbone	0.37	**0.73**	0.18	-0.28
Burning behind breastbone	0.23	**0.76**	0.00	0.40
Acid taste or regurgitation	**0.83**	0.10	-0.05	0.04
Sore throat or hoarseness due to heartburn or acid reflux	0.39	0.21	0.09	**0.81**
Difficulty sleeping due to heartburn or acid reflux	**0.53**	**0.61**	-0.02	0.20
Difficulty eating preferred foods due to heartburn or acid reflux	**0.82**	0.00	0.25	0.11
Difficulty working due to heartburn or acid reflux	**0.77**	0.31	0.05	0.04
Self-medication for heartburn or acid reflux	0.29	0.13	**0.92**	0.07

Numbers in bold indicate correlation coefficient ≥ 0.5.

For SF-36, the cumulative rate of the eight factors plus health transition item was 71.3%. Most items correlated with factors as expected (see Table 7), with particularly high correlations seen for role-physical and bodily pain dimensions. The physical functioning (PF) items were distributed into two dimensions; PFa included moderate to vigorous activities such as lifting or carrying groceries, climbing several flights of stairs and walking more than one mile, whereas PFb included less strenuous activities such as climbing one flight of stairs, bending, kneeling, walking one or several blocks, and bathing or dressing oneself. The social function dimension was unclear, distributing to mental health and role-emotional dimensions. In addition, two items from the vitality dimension, two from the mental health dimension and one from the physical functioning dimension were distributed into the general health dimension. The three role-emotional items showed a tendency towards distribution into the role-physical dimension, although the correlation coefficients were lower than those for distribution into the expected role-emotional dimension.

**Table 7:** Factor analysis matrix of SF-36 items in nine factors.

Item	GH	RP	PFa	PFb	MH	RE	BP	VT	HT
PF1	0.51	0.10	-0.03	**0.65**	-0.03	-0.01	0.06	0.10	0.08
PF2	0.33	0.18	0.12	**0.73**	0.12	0.12	-0.07	0.02	0.12
PF3	0.34	0.15	0.20	**0.72**	0.11	0.04	0.12	0.02	0.10
PF4	0.33	-0.02	0.32	**0.64**	0.14	0.04	0.06	-0.02	0.23
PF5	0.16	0.11	**0.81**	0.19	0.01	0.10	0.08	0.06	0.04
PF6	0.16	0.02	**0.57**	0.34	0.10	0.13	0.26	0.05	0.14
PF7	0.41	0.03	0.30	**0.56**	-0.05	0.01	-0.01	0.11	-0.23
PF8	0.36	0.04	0.49	0.46	0.06	-0.11	-0.07	-0.04	-0.15
PF9	0.13	0.13	**0.88**	0.08	0.12	-0.03	0.01	-0.02	-0.01
PF10	0.10	0.12	**0.89**	0.03	0.06	0.00	-0.06	0.00	-0.02
RP1	0.20	**0.91**	0.07	0.03	0.10	0.14	0.05	0.03	0.08
RP2	0.18	**0.90**	0.08	0.04	0.09	0.13	0.05	0.03	0.09
RP3	0.18	**0.88**	0.09	0.08	0.10	0.14	0.14	0.03	0.06
RP4	0.18	**0.82**	0.14	0.11	0.10	0.06	0.19	0.02	0.03
RE1	0.17	0.58	-0.01	0.07	0.13	**0.72**	0.04	0.02	-0.01
RE2	0.18	0.59	0.06	0.07	0.12	**0.72**	0.04	0.02	-0.02
RE3	0.13	0.48	0.07	0.09	0.09	**0.73**	0.05	0.05	-0.04
MH1	0.19	0.11	-0.03	0.06	**0.62**	0.13	0.18	0.15	-0.09
MH2	0.25	0.15	0.14	0.13	**0.64**	0.21	-0.06	0.08	0.15
MH3	0.57	0.05	0.03	0.07	0.37	-0.08	-0.03	-0.08	-0.25
MH4	0.20	0.12	0.18	0.02	**0.69**	0.11	-0.07	0.18	0.21
MH5	0.63	0.04	0.04	0.11	0.06	-0.01	-0.21	0.29	-0.17
VT1	0.76	0.10	0.05	0.27	0.19	-0.09	0.10	0.01	-0.17
VT2	0.78	0.13	0.05	0.25	0.17	-0.06	0.08	0.01	-0.15
VT3	0.27	0.05	0.05	0.04	0.13	0.04	0.08	**0.86**	0.05
VT4	0.37	0.10	0.02	0.10	0.19	-0.05	0.07	**0.75**	-0.03
SF1	0.23	0.27	0.16	-0.01	0.22	0.46	0.26	-0.18	0.07
SF2	0.27	0.18	0.08	0.06	0.64	-0.11	0.02	-0.07	-0.13
GH1	**0.59**	0.15	0.06	0.15	-0.06	0.10	0.16	0.00	0.43
GH2	**0.68**	0.10	0.02	0.03	0.10	0.12	0.05	0.13	0.13
GH3	**0.73**	0.07	0.19	-0.03	0.02	0.12	-0.01	0.02	0.08
GH4	**0.61**	0.05	-0.01	0.06	0.09	0.05	0.07	0.03	0.44
GH5	**0.74**	0.16	0.09	0.13	-0.03	0.14	0.16	0.06	0.18
BP1	0.20	0.24	0.07	0.04	0.04	0.08	**0.86**	0.05	0.09
BP2	0.20	0.32	0.07	0.07	0.03	0.06	**0.83**	0.09	0.04
HT	0.15	0.27	0.04	0.20	0.03	-0.06	0.09	-0.01	**0.68**

Numbers in bold indicate expected distribution with correlation coefficient ≥ 0.5. Underlined numbers indicate aberrant distribution. BP: bodily pain; GERD: gastroesophageal reflux disease; GH: general health; HT: health transition; MH: mental health; PF: physical function (PFa: strenuous activities; PFb: light activities); RE: role–emotional; RP: role–physical; SF: social function; SF-36: short form-36; VT: vitality.

The factor analysis showed that the construct validity of QOLRAD was not as good as expected, as items were not distributed to the appropriate dimensions (Table 8).

**Table 8:** Factor analysis matrix of QOLRAD items in five factors.

Item	Factors				
	1	2	3	4	5

Q4001	0.797				
Q4002	0.526			0.784	
Q4003	0.677				0.576
Q4004	0.874				
Q4005	0.808				
Q4006	0.852				
Q4007	0.879				
Q4008	0.829				
Q4009	0.826				
Q4010	0.903				
Q4011	0.867				
Q4012	0.912				
Q4013	0.890				
Q4014	0.812				
Q4015	0.931				
Q4016	0.736				
Q4017	0.879				
Q4018	0.954				
Q4019	0.940				
Q4020	0.516	0.794			
Q4021	0.796		0.557		
Q4022	0.825				
Q4023	0.938				
Q4024	0.954				
Q4025	0.930				

QOLRAD: Quality of Life in Reflux and Dyspepsia.

### Convergent validity

The RDQ GERD score was negatively correlated with all QOLRAD dimensions; correlations were statistically significant for the QOLRAD dimensions of food and drink problems (p = 0.037, correlation coefficient -0.28) and social functioning (p = 0.003, correlation coefficient -0.39). The RDQ GERD score was also significantly negatively correlated with all dimensions of SF-36 (p ≤ 0.001). SF-36 correlation coefficients ranged from -0.11 (social functioning) to -0.34 (bodily pain). Correlations were negative because health-related quality of life decreases as symptoms and their impact increase.

The RDQ GERD score correlated most strongly with bodily pain (the SF-36 dimension most impaired by GERD in previous studies), reflecting the fact that GERD is primarily a painful disease. All eight SF-36 dimensions were significantly correlated with the QOLRAD total score (p ≤ 0.001, correlation coefficients ranged from 0.16–0.29), supporting the construct validity of QOLRAD and SF-36.

## Discussion

This pilot study used several well-designed questionnaires, administered together, with the aim of developing and validating a methodology for the epidemiological study of GERD in China. Using a randomized, stratified, multi-stage cluster sampling technique, we validated Chinese translations of the SF-36, QOLRAD questionnaire, GERD Impact Scale and RDQ. In this study, the translated and adapted questionnaires demonstrated reproducibility and internal consistency within the methodology adopted, although responsiveness was not assessed. Each questionnaire had a test-retest reliability coefficient larger than 0.7 and a high Cronbach's alpha coefficient (≥ 0.8), suggesting good reliability. The construct validity of questionnaires was also credible in this survey, although the QOLRAD did not perform well in the factor analysis. This was likely to be due to linguistic and cultural translation problems: facilitators considered that some items were difficult to explain to respondents, particularly for those with a low level of education.

The sampling and administration techniques contributed substantially to the success of this study. By gaining the support of local residential communities, a high response rate of 86% was achieved, which is likely to prevent significant responder bias. The provision of assistance from trained facilitators helped avoid potential cultural and linguistic confusion, providing a relatively precise interpretation of the items in the questionnaire, and is recommended for future epidemiological studies using this survey instrument in order to ensure accuracy.

Chinese translations of the SF-36 have previously undergone psychometric validation among Chinese-speaking peoples in mainland China, the USA, Hong Kong and Taiwan [29,38-41]. These studies demonstrated satisfactory psychometric characteristics for SF-36 in these groups, while highlighting a level of cultural variation between Western and Chinese versions and between the different Chinese cultures. There is a tendency, also reflected in the current study, for the social functioning dimension to perform less well in China [29]; Li and colleagues have commented that this points to the Confucian ideology of collectivism in China, where it is socially unacceptable for Chinese to use 'sickness' as an excuse to avoid working or socializing [29].

In several previous studies vitality was more strongly associated with mental health than physical health [29,38-40], which may relate to traditional Chinese medicine, where fatigue associated with depression is conceptualized as a deficiency of vital energy or 'qi'. Although this was not the case in the current study, two items in the vitality dimension were more strongly distributed to general health. These issues illustrate the importance of examining the psychometric validity of instruments in different ethnic groups with cultural differences in language, values and perceptions of health.

This study has several limitations. Some subjects found the combined questionnaire too long and repetitive: a general information questionnaire, the RDQ, GERD Impact Scale, QOLRAD and SF-36 combined to make a total of 137 items and, on average, the questionnaire took about 20 minutes to complete. Responsiveness to change and known-groups validity were not assessed. Where construct validity was assessed, the different recall periods for individual questionnaires may have weakened convergent correlation results, while the short retest period may distort the reliability analysis where respondents remember their previous responses. The methodology was unable to sample migrant workers, who make up a significant portion of the Shanghai population, as they remain officially registered in their place of origin.

## Conclusion

The experience gained in this pilot study will inform a planned larger study of the epidemiology of GERD across mainland China, which will establish the wider prevalence of GERD symptoms in China using representative study populations and a standardized, well-validated methodology. The survey questionnaire will be reduced in length and simplified, and symptoms will be assessed using the RDQ with a longer recall period (4 weeks). The QOLRAD questionnaire will be removed from the survey, due to its relatively poor performance in the factor analysis. Ideally, responsiveness to change and known-groups validity should be studied to investigate further the validity of the survey instruments. Health-related quality of life will be evaluated using the SF-36, and sleep disturbance will be investigated using the Epworth Sleepiness Scale (ESS). Endoscopic examination of randomly sampled subjects would also be informative, to allow comparison with recent studies conducted in the West [42,43].

In summary, this study developed and tested a successful survey methodology for the epidemiological study of GERD in China. The questionnaires used demonstrated credible reliability and construct validity, supporting their use in larger epidemiological surveys of GERD in China, and allowing the results of this study to be extrapolated to the general population of East China.

## Competing interests

This study was supported by AstraZeneca R&D, Mölndal, Sweden. Writing support was provided by Chris Winchester and Claire Mulligan of Oxford PharmaGenesis and funded by AstraZeneca R&D, Mölndal, Sweden. Jia He has served as the director of the Department of Health Statistics, Second Military Medical University and WHO/TDR Clinical Data Management Center, Shanghai, China, and also served as a director of the Chinese Biomedicine Statistics Institute. Jia He has received research funding from the National Natural Science Foundation of China, WHO and Shanghai Natural Science Foundation. Saga Johansson is an employee of AstraZeneca R&D, Mölndal, Sweden, and Mari-Ann Wallander was an employee of AstraZeneca R&D, Mölndal, Sweden at the time of the study.

## Authors' contributions

YC and XY participated in the acquisition of data, analysis and interpretation of data, and drafting the article. XQM and RW participated in the analysis and interpretation of data, and drafting and critically revising the article. SJ and MAW participated in the conception and design of the study, and critically revising the article. JH made substantial contributions to the conception and design of the study, supervised all aspects of its implementation, and critically revised the article. All authors read and approved the final manuscript.

## Pre-publication history

The pre-publication history for this paper can be accessed here:



## Supplementary Material

Additional file 1 A pilot survey of GERD incidence in general population, China. English and Mandarin Chinese translations of a survey instrument for the study of gastroesophageal reflux disease (GERD) in China, including a general information questionnaire, the Reflux Disease Questionnaire, the Quality of Life in Reflux and Dyspepsia Questionnaire, and the GERD Impact Scale. Click here for file
