# Supplementary material for: Validation of a survey methodology for gastroesophageal reflux disease in China
Source: BMC Gastroenterol. 2008 Aug 21;8:37. doi: 10.1186/1471-230X-8-37 (PMC2533006; doi:10.1186/1471-230X-8-37)

胃食管反流病（GERD）人群患病情况上海预调查

A pilot survey of GERD incidence in general population, Shanghai

Q0001	调查点代码:<br>Surveyed site number:	□[1]
Q0002	调查对象编号:<br>Respondent ID:	□□□□
Q0003	是初次调查还是再次调查？ 1.初次 2.再次<br>Is this the initial or retest interview? 1.Initial 2. Retest	□
Q0003a	如果是再次调查，请指明两次调查间的间隔天数：(天)<br>If retest interview, indicate number of days between initial and retest: (Days)	□□
Q0004	调查员编号<br>Interviewer ID	□□
Q0005	调查员姓名<br>Interviewer name	____________[2]
Q0006	调查完成日期(年/月/日)<br>Date of finish(Year/Month/Day)	□□/□□/□□
Q0007	调查结果： 1.完成 2.部分完成 3.失访<br>Final result 1. Finished 2. Part finished 3. Missing	□□
Q0008	督导员审核日期(年/月/日)<br>Date of audit(Year/Month/Day)	□□/□□/□□
	督导员签字<br>Signature of Supervisor	____________

AstraZeneca R&D Mölndal

第二军医大学卫生统计学教研室

2005.10

---

[1] 每个空格处填写一个数字

[2] 请用大写英文字母填写

目录

Content

内容<br>Content	页码<br>Page
介绍<br>Introduction	2
知情同意书<br>Consent Form	3
第一部分：基本情况<br>Part I: General Information	4
第二部分：反流性疾病问卷调查<br>Part II: RDQ	6
第三部分：胃食管反流病影响量表<br>Part III: GERD Impact Scale (GIS)	7
第四部分：有烧心症状的病人问卷调查<br>Part IV: QOLRAD	8
第五部分：Rome II 组合问卷<br>Part V: Combined Rome II questionnaire	12
第六部分：SF-36 健康调查问卷<br>Part VI: SF-36	16
附录：胃食管反流病医学常识<br>Appendix: General knowledge about GERD	19

介绍

Introduction

胃食管反流病（**GERD)**是一种常见疾病。西方国家中大约 **17-38%**的成人至少每周出现过一次 **GERD** 的主要症状：烧心和/或反流；**4-9%**的人每天都有症状。在 **90** 年代，治疗过反流疾病的诊断数量以每年 **4-9%**的速度递增，并且这一数目还在继续增加。反流疾病患者患食管并发症的危险不断增加，如 **Barrett** 食管，食管粘膜肠化生，这些易导致食管腺癌的发生。另外，普遍认为，**GERD** 与广泛的食管外表现如哮喘，慢性咳嗽，声嘶相关，且对生活质量有明显影响。长期治疗的成本是很大的。然而，在中国，关于 **GERD** 的认识程度还相对较低，**GERD** 与食管外症状的联系还不清楚。我们还没有充分了解反流疾病的流行病学及其明显渐增的发病率。研究 **GERD** 的患病率/发病率及其相关症状/合并疾病是非常重要的，我们的目的就是进行一项大规模的流行病学调查以估计中国人口中 **GERD** 的人群患病率，及 **GERD** 的危险因素和合并疾病。此项预调查是整个计划的开始部分。

Gastroesophageal reflux disease (GERD) is a common disorder. Approximately 17 – 38% of adults in the Western countries experienced heartburn and/or acid regurgitation, the main symptoms of GERD, at least once a week; and 4 –9% have daily symptoms (1-3). The number of treated reflux disease diagnoses has increased by 5-10% annually in the 1990s and this growth continues. Patients with reflux disease are at increased risk of esophageal complications, such as Barrett’s esophagus, intestinal metaplasia of esophageal mucosa which predisposes to adenocarcinoma of the esophagus. In addition, GERD is recognized to be associated with broad extra-esophageal manifestation such as asthma, chronic cough, hoarseness and significant impact on the quality of life. The cost of long-term medication is substantial. However, awareness of GERD is relatively low in China and the link (s) between GERD and extra-esophageal symptoms is poorly recognized. The epidemiology of reflux disease and its apparently increasing incidence is not sufficiently known. To study the prevalence/incidence of GERD and its associated symptoms /co-morbidities is of much importance. Our aim is to perform a large epidemiology study to estimate the population prevalence of GERD in Chinese population, and the risk factors and co-morbidity of GERD. This pilot study is the initial part of the entire project.

AstraZeneca R&D Mölndal
第二军医大学卫生统计学教研室

2005.10

知情同意书

Consent Form

尊敬的参与者：

您好。

您已被随机地选为本次研究的调查对象，我们将对您作有关调查。本次研究是阿斯利康国际药业公司与第二军医大学卫生统计学教研室联合开展的一项预调查研究，由专业人员进行调查。您所提供的信息仅用于了解影响您健康的一些问题，整个调查约需 30 分钟。

您提供的信息不会泄漏给任何人，只用于研究目的。您的姓名、地址和其他个人信息将从调查表中删去，仅用代码与您的姓名和您的回答相联系，单从调查表不能认出是您本人。调查组在必要时可能会再次与您联系。

您的参与是自愿的，并且可以在调查过程中退出。您可以拒绝回答调查问卷中的任何一个问题。

在调查过程中，如果您有任何问题，可直接问调查员或直接与第二军医大学卫生统计学教研室的督导员联系。

签字表示您已了解您在本次调查中需要做的事情并愿意参加本次调查。

Dear Participant,

You have been randomly selected to be part of this survey and we would, therefore, like to interview you. This survey is a pilot study conducted by the AstraZeneca International Pharmaceutical Company and Department of Health Statistics, Second Military Medical University (SMMU), and will be carried out by professional interviewers. The information you provide will only be used to understand the main things that affect your health. The interview will take approximately 30 minutes.

The information you provide is totally confidential and will not be disclosed to anyone. It will only be used for research purposes. Your name, address, and other personal information will be removed from the questionnaire, and only a code will be used to connect your name and your answers without identifying you. The Survey Team may contact you again only if it is necessary to complete the information on the survey.

Your participation is voluntary and you can withdraw from the survey after having agreed to participate. You are free to refuse to answer any question that is asked in the questionnaire.

If you have any questions about this survey you may ask interviewer or contact Principal Investigator of Department of Health Statistics, SMMU..

Signing this consent indicates that you understand what will be expected of you and are willing to participate in this survey.

调查对象签名：　　　　　　　　　　日期：

Sign of respondent: ______________　　　　**Date:** ☐☐/☐☐/☐☐

联系电话：___________________

第一部分：基本情况

Part I: General Information)

		填写答案处 Answer
Q1001	姓名(请用汉语拼音填写)　Full name (Please fill with English)	______[3]
Q1002	性别: 1-女性　2-男性　Sex:: 1-Female　2-Male	☐[4]
Q1003	年龄（岁）　Age (yrs)	☐☐☐
Q1003a	如果记不清具体年龄，请选择您所在的年龄段：<br>1. 18~19 岁　2. 20~29 岁　3. 30~39 岁<br>4. 40~49 岁　5. 50~59 岁　6. 60~69 岁　7. 70+<br>If you don't know/don't want to tell me your age　could you tell me the age range:<br>1. 18~19yrs　2. 20~29yrs　3. 30~39yrs<br>4. 40~49yrs　5. 50~59ysr　6. 60~69ysr　7. 70+	☐
Q1004	体重(公斤)　Weight(kg)	☐☐☐
Q1005	身高(厘米)　Height(cm)	☐☐☐
Q1006	婚姻状况: 1-已婚　2-未婚<br>Current marital status: 1- Married　2-Unmarried	☐
Q1007	最高学历:　1-小学以下　2-小学　3-初中　4-高中　5-大专/大学　6-硕士　7-博士<br>The highest level of education:　1-Less than primary school　2-Primary school completed　3-Secondary school completed　4-High school (or equivalent) completed<br>5-College / pre-university / University completed　6-Master degree　7-Doctor degree	☐
Q1008	您总共接受过多少年学校教育(包括复读时间)?<br>How many years of school education have you completed (including repeated grades)?	☐☐
Q1009	民族(请用汉语拼音填写)　Nationality (Please fill with English)	______
Q1010	职业:　1-公务员　2-专业技术人员(军人除外)　3-工人<br>4-农、林、牧、渔业从业者　5-服务行业人员　6-在校学生　7-军人<br>Current job:　1-Government employee　2-Professional or technician　(excluding armed forces)　3-Blue-collar worker　4-Agricultural or fisheries Worker<br>5-Personal services, marketing, or sales　6-Student in school　7-Armed forces	☐
Q1011	家庭月收入:　1-1999 元以下　2-2000~4999 元　3-5000~9999 元　4-10000 元以上<br>Total income of family per month:　1-less than 1999 Yuan　2-2000~4999 Yuan<br>3-5000~9999 Yuan　4-10000 Yuan or above	☐
Q1012	吸烟情况:<br>1-不吸　2-1~5 支/天　3-6~10 支/天<br>4-11~15 支/天　5-16~20 支/天　6-21 支/天 以上<br>Do you currently smoke?<br>1-No　2-1~5pieces/day　3-6~10pieces/day<br>4-11~15pieces/day　5-16~20pieces/day　6-21pieces/day or above	☐
Q1012a	如果您吸烟，请说明您大概吸了多少年了？<br>If you smoke, for how many years are you have been smoking?	☐☐
Q1013	饮酒情况:　1-不饮酒　2.-每月少于 4 次　3-每周至少一次　4-每天至少一次<br>Do you currently drink?:　1-No　2-Less than 4 times per month<br>3-At least 1 time per week　4-At least 1 time per day	☐
Q1013a	如果您饮酒，请说明您大概饮酒多少年了？<br>If you drink, for how many years are you have been drinking?	☐☐
Q1014	请选择您平时进行体育运动的频率:<br>1-不运动　2-每月少于 4 次　3-每周至少一次　4-每天至少一次<br>How often do you take physical activities?<br>1-Never　2- Less than 4 times per month<br>3-At least 1 time per week　4-At least 1 time per day	☐

3 横线处请用大写字母填写。
4 每个空格处填写一个数字，带有小数者请四舍五入。

Q1015	总体来说，您的健康状况： 1-非常好 2-好 3-一般 4-差 5-很差<br>In general, how would you rate your health? 1-Very good 2-Good 3-Moderate 4-Bad 5-Very Bad	☐
Q1016	总体来说，您从事日常活动的能力： 1-非常好 2-好 3-一般 4-差 5-很差<br>Overall, how would you rate your daily activity? 1-Very good 2-Good 3-Moderate 4-Bad 5-Very Bad	☐
Q1017	总体来说，您从事工作的能力： 1-非常好 2-好 3-一般 4-差 5-很差<br>Overall, how would you rate your daily work?: 1-Very good 2-Good 3-Moderate 4-Bad 5-Very Bad	☐
Q1018	总体来说，您的精神状态： 1-非常好 2-好 3-一般 4-差 5.-很差<br>Overall, how would you rate your spirit? 1-Very good 2-Good 3-Moderate 4-Bad 5-Very Bad	☐
Q1019	总体来说，您的社会交往情况： 1-非常好 2-好 3-一般 4-差 5-很差<br>Overall, how would you rate your interpersonal activities? 1-Very good 2-Good 3-Moderate 4-Bad 5-Very Bad	☐
**Q1020**	你是否被医生诊断为以下情况/疾病？（如果是，最早做出这一诊断是在您多大年纪时？）<br>**Have you been diagnosed by a physician with any of the following conditions/diseases?？（If yes, at which age was it first diagnosed）**	年龄（岁）<br>**Age（yrs）**
Q1020a	高血压 Hypertension	☐☐
Q1020b	缺血性心脏病 Ischemic heart disease	☐☐
Q1020c	脑血管功能异常 Cerebrovascular disorder	☐☐
Q1020d	糖尿病 Diabetes	☐☐
Q1020e	慢性阻塞性肺病 COPD	☐☐
Q1020f	哮喘 Asthma	☐☐
Q1020g	肾功能异常 Renal disorder	☐☐
Q1020h	肝功能异常 Liver disorder	☐☐
Q1020i	风湿性关节炎 Rheumatoid arthritis	☐☐
Q1020j	骨关节炎 Osteoarthritis	☐☐
Q1020k	焦虑症 Anxiety	☐☐
Q1020l	抑郁症 Depression	☐☐
**Q1021**	您现在正在服下列药吗？如果有，请在后面写出已服用了多久<br>**Are you taking following drugs currently? If yes, please write the duration that you have taken them?**	时间(月)<br>**Duration(months)**
Q1021a	皮质类固醇 Corticosteroids	☐☐
Q1021b	荷尔蒙替代品 Hormone replacement	☐☐
Q1022	现在服用的其它药物一，名称： Others (name):	________[5]
Q1022a	服用了多少时间(月) Duration of treatment(months)	☐☐
Q1023	现在服用的其它药物二，名称： Others (name):	________
Q1023a	服用了多少时间(月) Duration of treatment(months)	☐☐
Q1024	现在服用的其它药物三，名称： Others (name):	________
Q1024a	服用了多少时间(月) Duration of treatment(months)	☐☐

[5] 如不知英文名，可用中文填写。

第二部分：反流性疾病问卷调查

Part II: RDQ

**Q2001**	**回想过去 7 天，你出现以下症状有多频繁?**<br>**0-无 1-每周少于 1 天 2-每周 1 天**<br>**3-每周 2~3 天 4-每周 4~6 天 5-每天**<br>**Thinking about your symptoms over the past 7 days, how often did you have the following?**<br>**0-No 1-Less than one day a week 2-One day a week 3-2-3days a weekb 4-4-6 days a week 5-Daily**	**填写答案处**<br>**Answer**
Q2001a	胸骨后感到灼热<br>A burning feeling behind your breastbone	☐[6]
Q2001b	胸骨后感到疼痛<br>Pain behind your breastbone	☐
Q2001c	上腹中心感到灼热<br>A burning feeling in the centre of the upper stomach	☐
Q2001d	上腹中心感到疼痛<br>A pain in the centre of the upper stomach	☐
Q2001e	口腔内有酸味<br>An acid taste in your mouth	☐
Q2001f	有东西从胃部向上移动而感到不适<br>Unpleasant movement of material upwards from the stomach	☐
**Q2002**	**回想过去 7 天，你认为以下症状出现时的程度如何?**<br>**0-无 1-很轻微 2-轻微**<br>**3-中度 4-较严重 5-严重**<br>**Thinking about symptoms over the past 7 days, how would you rate the following?**<br>**0-Did not have 1-Very mild 2-Mild**<br>**3-Moderate 4-Moderately severe 5-Severe**	
Q2002a	胸骨后感到灼热<br>A burning feeling behind your breastbone	☐
Q2002b	胸骨后感到疼痛<br>Pain behind your breastbone	☐
Q2002c	上腹中心感到灼热<br>A burning feeling in the centre of the upper stomach	☐
Q2002d	上腹中心感到疼痛<br>A pain in the centre of the upper stomach	☐
Q2002e	口腔内有酸味<br>An acid taste in your mouth	☐
Q2002f	有东西从胃部向上移动而感到不适<br>Unpleasant movement of material upwards from the stomach	☐

[6] 用问题中所给代码填写，若无请填“0”

第三部分：胃食管反流病影响量表（GIS）

Part III: GERD Impact Scale (GIS)

请考虑您在过去 2 周内的症状，并用所提供的选择项完成下列问题，每个问题只能填写一个选择项，您的回答没有对错之分。请确定您回答了每一个问题。

选择项：
1- 所有时间　2- 大多数时间　3- 一些时间　4- 从来没有

Please consider your symptoms over the past 2 weeks, and complete the following questions with provide selections. Each question only can be marked with one selection. There are no right or wrong answers. Please be sure to answer every question.

Selections:
1-All of the time　2-Some of the time　3-A little of the time　4-None of the time

**Q3001**	**在过去 2 周里，您有多经常地感觉到下列症状：**<br>**In the past 2 weeks, How often have you had the following symptoms:**	**填写答案处**<br>**Answer**
Q3001a	胸部或胸骨后疼痛<br>Pain in your chest or behind the breast bone	☐
Q3001b	胸部或胸骨后的烧灼感(即通常所说的烧心)<br>Burning sensation in your chest or behind the breast bone	☐
Q3001c	口腔内有反流或酸味<br>Regurgitations or acid taste in your mouth?	☐
Q3001d	与烧心或反酸有关的喉痛或喉咙嘶哑<br>Experienced a sore throat or hoarseness that is related to your heartburn or acid reflux?	☐
Q3002	您多经常地由于烧心或反酸而晚上难以很好地睡眠？<br>How often have you had difficulty getting a good night's sleep because of heartburn or acid reflux?	☐
Q3003	您有多经常地由于上述症状而无法吃、喝您所喜欢的东西？<br>How often have your symptoms prevented you from eating or drinking any of the foods you like?	☐
Q3004	您有多经常地由于上述症状而无法充分完成您的工作或日常活动?<br>How frequently have your symptoms kept you from being fully productive in your job or daily activities?	☐
Q3005	除了医生告诉您应该服用的药物外，您有多经常地服用额外的药物（如胃必治、乐得胃、泰胃美、西米替丁、雷尼替丁、法莫替丁等)?<br>How often do you take additional medication other than what the physician told you to take (such as Tums, Rolaids, Maalox)?	☐

第四部分：有烧心症状的病人问卷调查

Part IV: QOLRAD

以下一些问题询问您烧心或胃酸反流等症状所导致的感觉。烧心是指从胃部或下胸口走向颈部的烧灼感觉。胃酸反流是指带酸液体反流到喉咙或口腔。请尽量如实回答所有问题。在每条问题中，选出能最贴切描述您于过去一星期内的感觉的选择项。

On the following pages you will find some questions asking about how you have been feeling because of symptoms of heartburn or acid regurgitation. Heartburn is defined as a burning feeling rising from your stomach or lower chest up towards your neck. Acid regurgitation is defined as acid tasting liquid returning to your throat or mouth. Please answer all of these questions as honestly as you can. For each question, check the box which best describes how you have been feeling during the past week.

Q4001	在过去一星期内，你有多少时间会因烧心或胃酸反流而感到疲倦或疲劳？<br>1-全部时候 2-大部份时候 3-很多时候 4-有时候<br>5-很少时候 6-极少时候 7-完全没有<br>How often during the past week have you been feeling tired or worn out because of heartburn or acid regurgitation?<br>1-All of the time 2-Most of the time 3-Quite a lot of the time<br>4-Some of the time 5-A little of the time 6-Hardly any of the time<br>7-None of the time	☐
Q4002	在过去一星期内，你有多少时间会因烧心或胃酸反流而要避免弯曲身体？<br>1-全部时候 2-大部份时候 3-很多时候 4-有时候<br>5-很少时候 6-极少时候 7-完全没有<br>How often during the past week did you avoid bending over because of heartburn or acid regurgitation?<br>1-All of the time 2-Most of the time 3-Quite a lot of the time<br>4-Some of the time 5-A little of the time 6-Hardly any of the time<br>7-None of the time	☐
Q4003	在过去一星期内，你有多少烧心或胃酸反流的症状是因为饮食而造成？<br>1-非常多 2-很多 3-中等 4-有些 5-少许 6-极少 7-没有<br>During the past week, how much heartburn or acid regurgitation have you had because of eating or drinking?<br>1-A great deal 2-A lot 3-A moderate amount 4-Some 5-A little 6-Hardly any<br>7-None at all	☐
Q4004	在过去一星期内，你有多少时间会因烧心或胃酸反流而感到不适？<br>1-全部时候 2-大部份时候 3-很多时候 4-有时候<br>5-很少时候 6-极少时候 7-完全没有<br>How often during the past week have you felt generally unwell because of heartburn or acid regurgitation?<br>1-All of the time 2-Most of the time 3-Quite a lot of the time<br>4-Some of the time 5-A little of the time 6-Hardly any of the time<br>7-None of the time	☐
Q4005	在过去一星期内，你有多少时间会因烧心或胃酸反流而需要减少进食量？<br>1-全部时候 2-大部份时候 3-很多时候 4-有时候<br>5-很少时候 6-极少时候 7-完全没有<br>How often during the past week was it necessary to eat less than usual because of heartburn or acid regurgitation?<br>1-All of the time 2-Most of the time 3-Quite a lot of the time<br>4-Some of the time 5-A little of the time 6-Hardly any of the time<br>7-None of the time	☐
Q4006	在过去一星期内，你有多少时间会因烧心或胃酸反流而不能与家人或朋友进行活动?<br>1-全部时候 2-大部份时候 3-很多时候 4-有时候<br>5-很少时候 6-极少时候 7-完全没有	☐

	How often during the past week has heartburn or acid regurgitation kept you from doing things with family or friends?<br>1-All of the time 2-Most of the time 3-Quite a lot of the time<br>4-Some of the time 5-A little of the time 6-Hardly any of the time<br>7-None of the time	
Q4007	在过去一星期内，你有多少时间会因烧心或胃酸反流而导致精力缺乏？<br>1-全部时候 2-大部份时候 3-很多时候 4-有时候<br>5-很少时候 6-极少时候 7-完全没有<br>How often during the past week did you have a lack of energy because of heartburn or acid regurgitation?<br>1-All of the time 2-Most of the time 3-Quite a lot of the time<br>4-Some of the time 5-A little of the time 6-Hardly any of the time<br>7-None of the time	☐
Q4008	在过去一星期内，你有多少时间会因**烧心**或胃酸反流而在晚上难以安睡？<br>1-全部时候 2-大部份时候 3-很多时候 4-有时候<br>5-很少时候 6-极少时候 7-完全没有<br>How often during the past week have you had difficulty getting a good night’s sleep because of heartburn or acid regurgitation?<br>1-All of the time 2-Most of the time 3-Quite a lot of the time<br>4-Some of the time 5-A little of the time 6-Hardly any of the time<br>7-None of the time	☐
Q4009	在过去一星期内，你有多少时间会因**烧心**或胃酸反流而难以进食任何喜爱的食物或零食？<br>1-全部时候 2-大部份时候 3-很多时候 4-有时候<br>5-很少时候 6-极少时候 7-完全没有<br>How often during the past week has heartburn or acid regurgitation made it difficult to eat any of the foods or snacks you like?<br>1-All of the time 2-Most of the time 3-Quite a lot of the time<br>4-Some of the time 5-A little of the time 6-Hardly any of the time<br>7-None of the time	☐
Q4010	在过去一星期内，你有多少时间会因**烧心**或胃酸反流引起的睡眠缺乏而导致疲倦或疲劳?<br>1-全部时候 2-大部份时候 3-很多时候 4-有时候<br>5-很少时候 6-极少时候 7-完全没有<br>How often during the past week did you feel tired or worn out due to lack of sleep because of heartburn or acid regurgitation?<br>1-All of the time 2-Most of the time 3-Quite a lot of the time<br>4-Some of the time 5-A little of the time 6-Hardly any of the time<br>7-None of the time	☐
Q4011	在过去一星期内，你有多少时间会在晚上被**烧心**或胃酸反流弄醒而再难以入睡？<br>1-全部时候 2-大部份时候 3-很多时候 4-有时候<br>5-很少时候 6-极少时候 7-完全没有<br>How often during the past week did heartburn or acid regurgitation wake you up at night and prevent you from falling asleep again?<br>1-All of the time 2-Most of the time 3-Quite a lot of the time<br>4-Some of the time 5-A little of the time 6-Hardly any of the time<br>7-None of the time	☐
Q4012	在过去一星期内，你有多少时间会因**烧心**或胃酸反流而感到沮丧或苦恼？<br>1-全部时候 2-大部份时候 3-很多时候 4-有时候<br>5-很少时候 6-极少时候 7-完全没有<br>How often during the past week have you felt discouraged or distressed because of heartburn or acid regurgitation?<br>1-All of the time 2-Most of the time 3-Quite a lot of the time<br>4-Some of the time 5-A little of the time 6-Hardly any of the time<br>7-None of the time	☐
Q4013	在过去一星期内，你有多少时间会因**烧心**或胃酸反流而对食物失去兴趣？<br>1-全部时候 2-大部份时候 3-很多时候 4-有时候<br>5-很少时候 6-极少时候 7-完全没有<br>How often during the past week has heartburn or acid regurgitation made food seem unappealing to you?<br>1-All of the time 2-Most of the time 3-Quite a lot of the time<br>4-Some of the time 5-A little of the time 6-Hardly any of the time<br>7-None of the time	☐

Q4014	在过去一星期内，你有多少时间会因烧心或胃酸反流而感到无奈或烦燥?<br>1-全部时候 2-大部份时候 3-很多时候 4-有时候<br>5-很少时候 6-极少时候 7-完全没有<br>How often during the past week have you felt frustrated or impatient because of heartburn or acid regurgitation?<br>1-All of the time 2-Most of the time 3-Quite a lot of the time<br>4-Some of the time 5-A little of the time 6-Hardly any of the time<br>7-None of the time	☐
Q4015	在过去一星期内，你有多少时间会因烧心或胃酸反流而感到焦虑或不安？<br>1-全部时候 2-大部份时候 3-很多时候 4-有时候<br>5-很少时候 6-极少时候 7-完全没有<br>How often during the past week have you been anxious or upset because of heartburn or acid regurgitation?<br>1-All of the time 2-Most of the time 3-Quite a lot of the time<br>4-Some of the time 5-A little of the time 6-Hardly any of the time<br>7-None of the time	☐
Q4016	在过去一星期内，你有多少烧心或胃酸反流的症状，是因为进食了不能耐受的食物或零食？<br>1-非常多 2-很多 3-中等 4-有些 5-少许 6-极少 7-没有<br>During the past week, how much heartburn or acid regurgitation have you had because of having eaten foods or snacks you could not tolerate?<br>1-A great deal 2-A lot 3-A moderate amount 4-Some 5-A little 6-Hardly any<br>7-None at all	☐
Q4017	在过去一星期内，你有多少时间会因烧心或胃酸反流而对健康感到担心或忧虑？<br>1-全部时候 2-大部份时候 3-很多时候 4-有时候<br>5-很少时候 6-极少时候 7-完全没有<br>How often during the past week have you had any worries or fears about your health because of heartburn or acid regurgitation?<br>1-All of the time 2-Most of the time 3-Quite a lot of the time<br>4-Some of the time 5-A little of the time 6-Hardly any of the time<br>7-None of the time	☐
Q4018	在过去一星期内，你有多少时间会因烧心或胃酸反流而在早上起床时感到精神不振或睡眠不足?<br>1-全部时候 2-大部份时候 3-很多时候 4-有时候<br>5-很少时候 6-极少时候 7-完全没有<br>How often during the past week did you fail to wake up in the morning feeling fresh and rested because of heartburn or acid regurgitation?<br>1-All of the time 2-Most of the time 3-Quite a lot of the time<br>4-Some of the time 5-A little of the time 6-Hardly any of the time<br>7-None of the time	☐
Q4019	在过去一星期内，你有多少时间会因烧心或胃酸反流而感到易怒？<br>1-全部时候 2-大部份时候 3-很多时候 4-有时候<br>5-很少时候 6-极少时候 7-完全没有<br>How much during the past week has heartburn or acid regurgitation made you feel irritable?<br>1-All of the time 2-Most of the time 3-Quite a lot of the time<br>4-Some of the time 5-A little of the time 6-Hardly any of the time<br>7-None of the time	☐
Q4020	在过去一星期内，你有多少时间会因烧心或胃酸反流而必须避免进食某些食物或饮料？<br>1-全部时候 2-大部份时候 3-很多时候 4-有时候<br>5-很少时候 6-极少时候 7-完全没有<br>How often during the past week have you had to avoid certain food, beverages or drinks because of heartburn or acid regurgitation?<br>1-All of the time 2-Most of the time 3-Quite a lot of the time<br>4-Some of the time 5-A little of the time 6-Hardly any of the time<br>7-None of the time	☐
Q4021	在过去一星期内，你有多少时间会因烧心或胃酸反流而难以入睡？<br>1-全部时候 2-大部份时候 3-很多时候 4-有时候<br>5-很少时候 6-极少时候 7-完全没有<br>How often during the past week did you have trouble getting to sleep because of heartburn or acid regurgitation?<br>1-All of the time 2-Most of the time 3-Quite a lot of the time	☐

	4-Some of the time 5-A little of the time 6-Hardly any of the time<br>7-None of the time	
Q4022	在过去一星期内，你有多少时间因不知道**烧心**或胃酸反流出现的原因而这些症状仍经常出现，感到无奈?<br>1-全部时候 2-大部份时候 3-很多时候 4-有时候<br>5-很少时候 6-极少时候 7-完全没有<br>How often during the past week did you feel frustrated because the exact cause of your symptoms is not known and you still have so much heartburn or acid regurgitation?<br>1-All of the time 2-Most of the time 3-Quite a lot of the time<br>4-Some of the time 5-A little of the time 6-Hardly any of the time<br>7-None of the time	☐
Q4023	在过去一星期内，你有多少时间会因**烧心**或胃酸反流而难以与家人或朋友进行社交？<br>1-全部时候 2-大部份时候 3-很多时候 4-有时候<br>5-很少时候 6-极少时候 7-完全没有<br>How often during the past week did you have difficulty socializing with family or friends because of heartburn or acid regurgitation?<br>1-All of the time 2-Most of the time 3-Quite a lot of the time<br>4-Some of the time 5-A little of the time 6-Hardly any of the time<br>7-None of the time	☐
Q4024	在过去一星期内，你有多少时间会因**烧心**或胃酸反流而无法进行日常活动（包括家庭以外的工作及家务）？<br>1-全部时候 2-大部份时候 3-很多时候 4-有时候<br>5-很少时候 6-极少时候 7-完全没有<br>How often during the past week were you unable to carry out your daily activities (including both work outside the home and house work)due to heartburn or acid regurgitation?<br>1-All of the time 2-Most of the time 3-Quite a lot of the time<br>4-Some of the time 5-A little of the time 6-Hardly any of the time<br>7-None of the time	☐
Q4025	在过去一星期内，你有多少时间会因**烧心**或胃酸反流而无法进行一般体力活动(包括运动、休闲活动及在家庭以外的活动）？<br>1-全部时候 2-大部份时候 3-很多时候 4-有时候<br>5-很少时候 6-极少时候 7-完全没有<br>How often during the past week were you unable to carry out your normal physical activities (including sport, leisure activities and moving around outside the home)due to heartburn or acid regurgitation?<br>1-All of the time 2-Most of the time 3-Quite a lot of the time<br>4-Some of the time 5-A little of the time 6-Hardly any of the time<br>7-None of the time	☐

第五部分：ROME II 组合问卷

Part V: Combined ROME II questionnaire

问题 Question		答案(请在对应的方框内打“√”) Answer		
**食道症状 Esophageal Disorders**				
Q5001	在过去 3 个月内，未做吞咽动作时，你是否*经常*[7]感到喉咙内有肿物？<br>In the last 3 months, did you often* get the feeling of a lump in your throat when you were not swallowing?	☐0 无或极少 No or rarely<br>☐1 是 Yes	⟶	直接跳至 Q5003。Skip to question Q5003。
Q5002	进食或饮水时，有否感到吞咽困难或疼痛？<br>When you are eating or drinking, is it difficult to swallow, or does it hurt to swallow?	☐0 无或极少 No or rarely<br>☐1 是 Yes		
Q5003	在过去 3 个月内，是否*经常有*食物反流至口中、经再咀嚼，然后吐出或再次吞下？<br>In the last 3 months, did you often* bring up food, chew it again, and either spit it out or re-swallow it?	☐0 无或极少 No or rarely<br>☐1 是 Yes	⟶	直接跳至 Q5006。Skip to question Q5006。
Q5004	在出现食物反流现象期间，你是否曾呕吐或感到胃部不适？<br>At these times, did you vomit or feel sick to your stomach?	☐0 无或极少 No or rarely<br>☐1 是 Yes		
Q5005	食物变酸后，是否不再出现食物反流？<br>Do you stop bringing up food when the food turns sour (acidic)?	☐0 无或极少 No or rarely<br>☐1 是 Yes		
Q5006	在过去 3 个月内，你是否*经常*感到胸部中央部位疼痛（但并不是由心绞痛或心脏病发作引致）？<br>In the last 3 months, did you often* have pain in the middle of your chest (that is not due to angina or a heart attack)?	☐0 无或极少 No or rarely<br>☐1 是 Yes	⟶	直接跳至 Q5008。Skip to question Q5008。
Q5007	当感觉食物下咽受阻时，是否有这种胸痛出现？<br>Did this chest pain occur when it felt like food got stuck going down?	☐0 无或极少 No or rarely<br>☐1 是 Yes		
Q5008	在过去 3 个月内，你是否*经常*有胃灼热感、胸部烧灼痛或不适？<br>In the last 3 months, did you often* have heartburn, a burning pain or discomfort in your chest?	☐0 无或极少 No or rarely<br>☐1 是 Yes		
Q5009	在过去 3 个月内，你是否*经常*在吞咽后出现困难（固体或液体黏在胸部，或无法正常下咽）？<br>In the last 3 months, did you often* have difficulty after swallowing (solid or liquids sticking in your chest, or passing down abnormally)?	☐0 无或极少 No or rarely<br>☐1 是 Yes		
**胃十二指肠症状 Gastroduodenal Disorders**				
Q5010	在过去 3 个月内， 你是否*经常*有上腹中央部位（肚脐上方或胃部）的不适或疼痛？<br>In the last 3 months, did you often* have discomfort or pain centered in your upper abdomen (above your belly button, or the pit of your stomach)?	☐0 无或极少 No or rarely<br>☐1 是 Yes	⟶	直接跳至 Q5015。Skip to question Q5015。

[7]*经常*指过去 3 个月内至少有 3 周（每周至少 1 日）出现症状。

Q5011	下列描述中哪项最符合你的症状或不适？<br>Check your best description of this symptom, or the one that bothers you more .	☐1 上腹或胃部的*疼痛* Pain in your upper abdomen or stomach<br>☐2 上腹或胃部的*不适*（并非疼痛）Discomfort (that is not painful) in your upper abdomen or stomach	⟶	直接跳至 Q5013。
Q5012	如果你感到不适，以下哪一项描述最符合你的不适？（*请选取所有适用者*）<br>If you have discomfort, which of thefollowing describe your discomfort?(check all that apply)	☐1 恶心 Nausea<br>☐2 腹胀（上腹饱胀的感觉）Bloating (a sensation of upper abdominal swelling)<br>☐3 进食极少量食物后即感到饱胀 Feeling full after eating very little<br>☐4 以上均不符合 None of the above		
Q5013	你的上腹不适或疼痛在排便后是否通常会得到改善或消失？<br>Does your upper abdominal discomfort or pain usually get better or stop after you have a bowel movement?	☐0 无或极少 No or rarely<br>☐1 是 Yes		
Q5014a	当上腹不适或疼痛发生时，你(习惯/经常)的排便次数是否会有改变（较平常多或少）？<br>When the upper abdominal discomfort or pain starts, do you usually have a change in your usual number of bowel movements (either more or fewer)?	☐0 无或极少 No or rarely<br>☐1 是 Yes		
Q5014b	当上腹不适或疼痛发生时，你的粪便是否通常会更软或更硬？<br>When the upper abdominal discomfort or pain starts, do you usually have either softer or harder stools than usual?	☐0 无或极少 No or rarely<br>☐1 是 Yes		
Q5015	在过去 3 个月内， 你是否*经常**打嗝或嗳气？<br>In the last 3 months, did you often* burp or belch?	☐0 无或极少 No or rarely<br>☐1 是 Yes	⟶	直接跳至 Q5017。 Skip to question Q5017。
Q5016	你是否曾吞咽空气以帮助打嗝？<br>Did you swallow air to help you belch?	☐0 无或极少 No or rarely<br>☐1 是 Yes		
Q5017	在过去 3 个月内， 你是否经常呕吐（每周至少 3 日）？<br>In the last 3 months, did you have frequent episodes of vomiting (on at least 3 separate days in each week)?	☐0 无或极少 No or rarely<br>☐1 是 Yes	⟶	直接跳至 Q5020。 Skip to question Q5020。
Q5018	在此期间， 是否曾经强迫自己呕吐？<br>During these episodes, did you make yourself vomit?	☐0 无或极少 No or rarely<br>☐1 是 Yes	⟶	直接跳至 Q5020。 Skip to question Q5020。
Q5019	你是否因正在服用的药物或患有其它疾病而出现呕吐？<br>Were you vomiting because of a medication you were taking or another medical condition that you had?	☐0 无或极少 No or rarely<br>☐1 是 Yes		
**排便症状 Bowel Disorders**				
Q5020	在过去 3 个月内， 你是否*经常*感到腹部不适或疼痛？<br>In the last 3 months, did you often* have discomfort or pain in your abdomen?	☐0 无或极少 No or rarely<br>☐1 是 Yes	⟶	直接跳至 Q5024。 Skip to question Q5024。
Q5021	你的不适或疼痛是否会在排便后改善或消失？<br>Does your discomfort or pain get better or stop after you have a bowel movement?	☐0 无或极少 No or rarely<br>☐1 是 Yes		

Q5022	腹部不适或疼痛发生时，你平常的排便次数是否会有改变（较平常多或少）？<br>When the discomfort or pain starts, do you have a change in your usual number of bowel movements (either more or fewer)?	☐0<br>☐1	无或极少 No or rarely<br>是 Yes		
Q5023	当腹部不适或疼痛发生时，你的粪便是否会较平常更软或更硬？<br>When the discomfort or pain starts, do you have either softer or harder stools than usual?	☐0<br>☐1	无或极少 No or rarely<br>是 Yes		
Q5024	在过去 3 个月内， 至少四分之一（1/4）的时间（可连续数日或不连续），出现以下任何症状？（*请选取所有适用者*）<br>Have you had any of the following symptoms at least one-fourth (1/4) of the time (occasions or days) in the last 3 months? (check all that apply)	☐1<br>☐2<br>☐3<br>☐4<br>☐5<br>☐6<br>☐7<br>☐8<br>☐9<br>☐10<br>☐11	每周排便少于3次（0－2次）Fewer than three bowel movements a week (0–2)<br>每日排便多于3次（4次或以上）More than three bowel movements a day (4 or more)<br>粪便坚硬或呈颗粒状 Hard or lumpy stools<br>粪便稀烂、呈糊状或呈水状 Loose, mushy or watery stools<br>排便费力 Straining during a bowel movement<br>想排便时有必须马上冲到厕所的急迫感 Having to rush to the toilet to have a bowel movement<br>排便后感到粪便尚未排净 Feeling of incomplete emptying after a bowel movement<br>排便时有黏液排出 Passing mucus (slime) during a bowel movement<br>腹胀、胀气或胀满 Abdominal fullness, bloating or swelling<br>排便时感到粪便难以排出（即被阻塞感）A sensation that the stool cannot be passed (i.e., blocked) when having a bowel movement<br>需要挤压臀部或阴部四周以助排便完成 A need to press on or around your bottom or vagina to try to remove stool in order to complete the bowel movement		
Q5025	在过去 3 个月内，你排出稀烂便、糊状便或水状便的次数是否多于总排便次数的四分之三 (3/4)？<br>In the last 3 months, did you have loose, mushy, or watery stools, during more than three quarters (3/4) of your bowel movements?	☐0<br>☐1	无或极少 No or rarely<br>是 Yes		
**腹痛症状 Functional Abdominal Pain**					
Q5026	在过去 6 个月内， 你是否总是（持续地）或大部份时间（接近持续地）感到腹痛？（*对女性而言，这种腹痛不是指与月经周期有关的腹痛*）<br>In the last 6 months, did you have pain in your abdomen all the time (continuously) or most of the time (nearly continuously)? (If you are female, this should not be related to your menstrual cycle or period)	☐0<br>☐1	无或极少 No or rarely<br>是 Yes	⟶	直接跳至Q5028。Skip to question Q5028。
Q5027	这种疼痛是否限制或约束了你工作或参与社交活动的能力？<br>Has this pain limited or restricted your ability to work or go to social events?	☐0<br>☐1	无或极少 No or rarely<br>是 Yes		
**胆道症状 Biliary Disorders**					
Q5028	在过去 1 年内， 上腹中央或右边部位是否出现过任何持续的严重疼痛？<br>In the last year, did you have any severe steady pain in the middle or right side of your upper abdomen?	☐0<br>☐1	无或极少 No or rarely<br>是 Yes	⟶	直接跳至Q5033。Skip to question Q5033。
Q5029	该疼痛是否持续 30 分钟或以上？<br>Did the pain last 30 minutes or more?	☐0<br>☐1	无或极少 No or rarely<br>是 Yes		

Q5030	该疼痛是否令你无法进行一般日常活动，或驱使你求医？<br>Did the pain keep you from your usual daily activities, or cause you to see a doctor?	☐0<br>☐1	无或极少 No or rarely<br>是 Yes		
Q5031	你的胆囊是否已经被切除？<br>Have you had your gallbladder removed?	☐0<br>☐1	无或极少 No or rarely<br>是 Yes	⟶	直接跳至 Q5033。Skip to question Q5033。
Q5032	自切除胆囊以来， 你的上腹中央或右边部位是否有过任何严重或持续性的疼痛？<br>Did you have any severe or steady pain in the middle or right side of your upper abdomen since your gallbladder was removed?	☐0<br>☐1	无或极少 No or rarely<br>是 Yes		
**门直肠症状 Anorectal Disorders**					
Q5033	在过去 1 年内，当出现便秘或腹泻时，你是否曾经在 1 个月内 1 次以上意外地渗出或排出粪便？<br>In the last year, when you had constipation or diarrhea, did you accidentally leak or pass stool for more than one occasion in a month?	☐0<br>☐1	无或极少 No or rarely<br>是 Yes	⟶	直接跳至 Q5035。Skip to question Q5035。
Q5034	你意外地排出多少粪便？你会形容为…<br>How much stool did you accidentally lose? Would you say . . .	☐1<br>☐2	小量（弄污内裤） A small amount (it stains underwear)<br>中等或大量（2茶匙或以上） A moderate or large amount (2 teaspoons or more)		
Q5035	在过去 1 年内，你是否有 1 次以上感到肛门管腔或直肠内隐隐作痛或受压？<br>In the last year, did you have more than one episode of aching pain or pressure in the anal canal or rectum?	☐0<br>☐1	无 No<br>是 Yes	⟶	直接跳至 Q5038。Skip to question Q5038。
Q5036	在过去 3 个月内，这种疼痛是否经常或持续出现？<br>Did this pain occur frequently or continuously in the last 3 months?	☐0<br>☐1	无 No<br>是Yes		
Q5037	以下两句陈述中，哪一句较贴切地描述了你肛门管腔或直肠内的持续疼痛、阵痛或受压的感觉？<br>Which of the following 2 statements better describes the aching, pain, or pressure that you had in the anal canal or rectum?	☐1<br>☐2	持续数秒至数分钟不等，然后完全消失 Lasts from seconds to minutes and disappears completely<br>持续 20 分钟以上，长达数日或更久 Lasts more than 20 minutes and up to several days or longer		
Q5038	在过去 3 个月内，你在排便时是否…<br>（*请选择所有合适的描述*）<br>In the last 3 months, when you were having bowel movements, did you. . .(check all that apply)	☐1<br>☐2<br>☐3<br>☐4	至少四分之一的时间感到好像必须用力才能排出粪便Feel as if you had to strain to pass your stool on at least one quarter of the time<br>至少四分之一的时间感到好像未能排清净直肠内的粪便 Feel as if you were unable to empty the rectum at least one quarter of the time<br>至少四分之一的时间难以放松或让粪便自然排出 Have difficulty relaxing or letting go to allow the stool to come out at least one quarter of the time<br>以上均不符合 None of the above		

第六部分：SF-36 健康调查问卷

Part VI: SF-36

本调查涉及你对自身健康的观点。这些信息将有助于追踪你从事日常活动的能力及自身感觉。请回答所有问题，在方框内填下你所选择的的数字。如果你对答案不确定，请给出你认为最接近的答案。

This survey asks for your views about your health. This information will help keep track of how you feel and how well you are able to do your usual activities.Answer every question by marking the answer as indicated. If you are unsure about how to answer a question, please give the best answer you can.

Q6001	总的来说，你认为你的健康状况： 1-棒极了 2-很好 3-好 4-过得去 5-糟糕<br>In general, would you say your health is: 1-Excellent 2-Very good 3-Good 4-Fair 5-Poor	☐
Q6002	与一年前相比，你如何评价现在的健康状况？<br>1-比一年前好多了 2-比一年前好一点 3-和一年前差不多<br>4-比一年前差一点 5-比一年前差多了<br>Compared to one year ago, how would you rate your health in general now?<br>1-Much better now than one year ago 2-Somehwat better now than one year ago 3-About the same as one year ago 4-Somewhat worse<br>now than one year ago 5-Much worse now than one year ago	☐
**Q6003**	下列项目是你平常在一天中可能做的事情。你现在的健康限制你从事这些活动吗？如果是的话，程度如何？<br>**选择项:**<br>**1-是，很受限 2-是，稍受限 3-不，完全不受限**<br><br>The following items are about activities you might do during a typical day. Does your health now limit you in these activities? Is so, how much?<br>**Selection:**<br>**1-Yes, limited a lot 2-Yes, limited a little 3-No, not limited at all**	
Q6003a	**高强度活动**，如跑步、举重物、参与剧烈运动：<br>Vigorous activities, such as running, lifting heavy objects, participating in strenuous sports:	☐
Q6003b	**中等度活动**，如移动桌子，推动真空吸尘器（或拖地板）、打保龄球、打高尔夫球（或打太极拳）：<br>Moderate activities, such as moving a table, pushing a vacuum cleaner, bowling, or playing golf:	☐
Q6003c	举或搬运杂物：<br>Lifting or carrying groceries:	☐
Q6003d	爬**数**层楼梯：<br>Climbing several flights of stairs:	☐
Q6003e	爬**一**层楼梯：<br>Climbing one flight of stairs:	☐
Q6003f	弯腰、屈膝：<br>Bending, kneeling, or stooping:	☐
Q6003g	步行 **1500 米以上**：<br>Walking more than a mile:	☐
Q6003h	步行**几个路口**：<br>Walking several blocks:	☐
Q6003i	步行**一个路口**：<br>Walking one block:	☐
Q6003j	自己洗澡或穿衣：<br>Bathing or dressing yourself:	☐

**Q6004**	**在过去 4 周，你是否因为生理健康原因，在工作或从事其他日常活动时有下列问题？**<br>**During the past 4 weeks, have you had any of the following problems with your work or other regular daily activities as a result of your physical health?**	
Q6004a	减少了工作或从事其他活动的**时间**：1-是 2-否<br>Cut down on the amount of time you spent on work or other activities: 1-Yes 2-No	☐
Q6004b	减少了工作**量**或活动**量**：1-是 2-否<br>Accomplished less than you would like：1-Yes 2-No	☐
Q6004c	从事工作或其他活动的**种类**受限：1-是 2-否<br>Were limited in the kind of work or other activities.1-Yes 2-No	☐
Q6004d	从事工作或其他活动有**困难**（例如，费劲）：1-是 2-否<br>Had difficulty performing the work or other activities (for example, it took extra effort): 1-Yes 2-No	☐
**Q6005**	**在过去 4 周，你是否因为任何情感问题（如感到抑郁或焦虑），在工作或从事其他日常活动时有下列问题？**<br>**During the past 4 weeks , have you had any of the following problems with your work or other regular daily activities as a result of any emotional problems (such as feeling depressed or anxious)**	
Q6005a	减少了工作或从事其他活动的**时间**：1-是 2-否<br>Cut down on the amount of time you spent on work or other activities: 1-Yes 2-No	☐
Q6005b	减少了工作**量**或活动**量**：1-是 2-否<br>Accomplished less than you would like：1-Yes 2-No	☐
Q6005c	不能象平常那么专心地从事工作或其他活动：1-是 2-否<br>Didn’t do work or other activities as carefully as usual：1-Yes 2-No	☐
Q6006	在过去 4 周，你的生理健康或情感问题在何种程度上干扰了你与家人、朋友、邻居、或团体的正常社会活动？ 1-完全没有 2-轻度 3-中度 4-重度 5-极度<br>During the past 4 weeks, to what extent has your physical health or emotional problems interfered with your normal social activities with family, friends, neighbors, or groups?<br>1-Not at all 2-Slightly 3-Moderately 4-Quite a bit 5-Extremely	☐
Q6007	在过去 4 周，你经受了多少躯体疼痛？<br>1-完全没有 2-很轻微 3-轻微 4-中等 5-严重 6-极严重<br>How much bodily pain have you had during the past 4 weeks?<br>1-None 2-Very mild 3-Mild 4-Moderate 5-Severe 6-Very severe	☐
Q6008	在过去 4 周，疼痛在多大程度上干扰了你的正常工作（包括户外工作和家务劳动）？<br>1-完全没有 2-一点点 3-中度 4-重度 5-极度<br>During the past 4 weeks, how much did pain interfere with your normal work (including both work outside the home and housework)?<br>1-Not at all 2-A little bit 3-Moderately 4-Quite a bit 5-Extremely	☐
**Q6009**	这些问题将问及你在过去4周的感觉和情感体验。对每一问题，请给出与你想法最接近的一个答案。在过去4周，**有多少时间…**<br>**选择项:**<br>**1-所有时间 2-绝大多数时间 3-很多时间 4-一些时间 5-一点时间 6-没有时间**<br>These questions are about how you feel and how things have been with you during the past 4 weeks. For each question, please give the one answer that comes closest to the way you have been feeling.<br>**How much of the time during the past 4 weeks**<br>**Selection:**<br>**1- All of the time 2-Most of the time 3-A good bit of the time**<br>**4-Some of the time 5-A little of the time 6-None of the time**	
Q6009a	你觉得干劲十足？<br>Did you feel full of pep?	☐
Q6009b	你是一个非常紧张的人？<br>Have you been a very nervous person?	☐
Q6009c	你感到情绪低落、沮丧，怎么也快乐不起来？<br>Have you felt so down in the dumps that nothing could cheer you up?	☐
Q6009d	你觉得平静、安适？<br>Have you felt calm and peaceful?	☐
Q6009e	你觉得精力旺盛？<br>Did you have a lot of energy?	

Q6009f	你感到闷闷不乐、心情忧郁？<br>Have you felt downhearted and blue?	☐
Q6009g	你觉得累极了？<br>Did you feel worn out?	☐
Q6009h	你是一个快乐的人？<br>Have you been a happy person?	☐
Q6009i	你觉得疲劳？<br>Did you feel tired?	☐
Q6010	在过去4周，有多少时间你的社会活动（如访问朋友，亲戚等）受你的生理健康或情感问题的影响动？1-所有时间 2-绝大多数时间 3-一些时间 4-一点时间 5-没有时间<br>During the past 4 weeks, how much of the time has your physical health or emotional problems interfered with your social activities (like visiting with friends, relatives, etc.)?<br>1-All the time 2-Most of the time 3-Some time 4-A little time 5-Non of the time	☐
**Q6011**	**下列每一种情形与你实际情况符合的程度如何?**<br>**How TRUE or FALSE is each of the following statements for you?**	
Q6011a	和其他人相比，我似乎更容易生病：<br>1-全部符合 2-大部分符合 3-不知道 4-大部分不符合 5-全部不符合<br>I seem to get sick a little easier than other people<br>1-Definitely true 2-Mostly true 3-Don’t know 4-Mostly false 5-Definitely false	☐
Q6011b	我和我认识的人一样健康：<br>1-全部符合 2-大部分符合 3-不知道 4-大部分不符合 5-全部不符合<br>I am as healthy as anybody I know<br>1-Definitely true 2-Mostly true 3-Don’t know 4-Mostly false 5-Definitely false	☐
Q6011c	我预计我的健康状况将变得更差：<br>1-全部符合 2-大部分符合 3-不知道 4-大部分不符合 5-全部不符合<br>I expect my health to get worse<br>1-Definitely true 2-Mostly true 3-Don’t know 4-Mostly false 5-Definitely false	☐
Q6011d	我的身体棒极了：<br>1-全部符合 2-大部分符合 3-不知道 4-大部分不符合 5-全部不符合<br>My health is excellent<br>1-Definitely true 2-Mostly true 3-Don’t know 4-Mostly false 5-Definitely false	☐

胃食管反流病医学常识

胃食管反流病(gastroesophageal reflux disease, GERD)是较常见的食管疾病。主要是由于胃内容物反流入食管，刺激食管粘膜而引起的炎症。酸（碱）反流导致的食管粘膜破损称为反流性食管炎(reflux esophagitis, RE)。正常人食管下端括约肌在不进行吞咽活动的时候是紧闭的，防止胃内容物向食管返流，但在一些诱因的作用下，此处括约肌不能正常地关闭，从而导致酸性的胃液或碱性的肠液反流入食管，并刺激、腐蚀食管粘膜，就引起胃食管反流病。

[临床表现]

胃食管反流病的主要症状是烧心（胸骨后烧灼感或烧灼样疼痛）、反酸和吞咽困难。

烧心常与姿势有关，故又叫做“姿势性烧心”，多在屈曲弯腰、咳嗽、用力排便、头低位仰卧或侧卧时出现。

胃内酸性容物反流至咽部或口腔时，会感到酸呛不适，这就是反酸；当炎症引起食管痉挛或食管瘢痕狭窄时，可出现吞咽困难，甚至吞咽疼痛，严重时可出现慢性食管出血。

当精神紧张或进食酸性果汁、高脂肪饮食、辛辣刺激性饮食以及吸烟、饮酒、服用咖啡因、巧克力，还有使用阿托品、普鲁本辛、654—2、巅茄片以及阿斯匹林等药物时，都会诱发或加重以上症状，而当直立和服用制酸药后；可减轻症状。

[诊断]

GERD 的诊断：（1）有明显的反流症状；（2）内镜下可能有反流性食管炎的表现；（3）过多胃食管返流的证据 。

RE 的诊断标准：有典型的 GERD 症状，如明显烧心、反酸、胸骨后灼痛等，无报警症状者需具备下列 RE 的证据:

(1) RE 的内镜诊断及分级（见表 1）。

表 1　RE 的内镜诊断与分级

分级	内镜下表现	积分
0	正常（可有组织学改变）	0
I	呈点状或条状发红、糜烂，无融合现象	1
II	有条状发红、糜烂，并有融合，但非全周性	2
III	病变广泛，发红、糜烂融合呈全周性，或溃疡	3

（2）RE的基本病理改变是：①食管鳞状上皮增生，包括基底细胞增生超过3层和上皮延伸；②粘膜固有层乳头向表面延伸，达上皮层厚度的2/3，浅层毛细血管扩张、充血和（或）出血；③上皮层内中性粒细胞和淋巴细胞浸润；④粘膜糜烂或溃疡形成，炎症细胞浸润，肉芽组织形成和（或）纤维化；⑤齿状线上>3cm出现Barrett食管改变。RE的病理分级见表2。

反流性食管炎时，可有鳞状上皮细胞假上皮瘤性增生，纤维母细胞和血管内皮细胞增生，伴一定程度的细胞异型性，应防止误诊为癌或肉瘤。

表2　反流性食管炎病理分级

病变	分级		
	轻度	中度	重度
鳞状上皮增生	＋	＋	＋
粘膜固有层乳头延伸	＋	＋	＋
上皮细胞层内炎症细胞浸润	＋	＋	＋
粘膜糜烂	－	＋	－
溃疡形成	－	－	＋
Barrett 食管改变	－	－	＋/－

（3）GERD的动力诊断依据

根据24h食管 pH监测的有关参数测算酸反流计分，>15分为阳性。15～50分为轻度GERD，51～100分为中度GERD，>100分为重度GERD。碱反流可用24h胆汁监测仪(Bilitec-2000)测定。

（4）质子泵抑制剂(PPI)试验

使用奥美拉唑（商品名：洛赛克）20 mg bid，共 7 天，如患者症状消失或显著好转，提示为明显的酸相关性疾病，在除外消化性溃疡等疾病后，应考虑 RE 的诊断。

GERD 及 RE 的临床分级:

以食管粘膜的内镜下表现作为判断 RE 级别的依据。内镜下食管粘膜 0 级为正常，Ⅰ级为轻度 RE，Ⅱ级为中度 RE，Ⅲ级为重度 RE；其中 0～Ⅰ级为轻度 GERD。

[治疗]

1. 治疗目的：①减轻或消除症状；②防治并发症；③预防复发。
2. 一般治疗：嘱患者抬高床头，避免精神刺激，少食多餐，戒烟酒，低脂、低糖清淡饮食，避免饱食。
3. 药物治疗：①PPI 口服：如奥美拉唑 20mg bid，疗程 8 周，维持量每日 10～20mg，至少 6 个月；②H2 受体阻制剂（H2RA）：如西米替丁、雷尼替丁、法莫替丁等；③促动力药：西沙必利 10mg tid 或 qd，并维持治疗。

根据临床分级，轻度GERD及RE可单独选用PPI、促动力药或H_2RA；中度GERD及RE宜采用PPI或H_2RA和促动力药联用；重度GERD及RE宜加大PPI口服剂量，或PPI与促动力药联用。

4．外科治疗：内科正规治疗无效或有并发症者可考虑外科手术治疗。

5．疗效分级：按内镜复查的积分判断疗效，内镜积分为 0 分者为痊愈；内镜积分减少 2 分者为显效；积分减少 1 分者为有效；积分无变化或增加 1 分以上者为无效。

资料来源：中华医学会消化病学分会

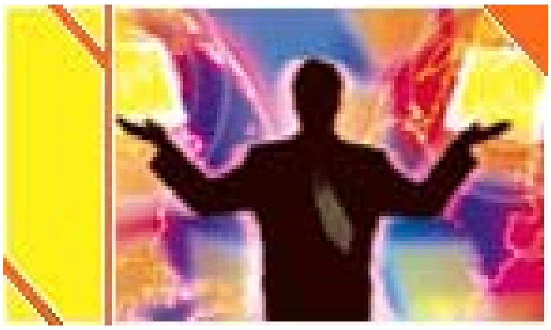

Supplement: Additional file 1 — A pilot survey of GERD incidence in general population, China. English and Mandarin Chinese translations of a survey instrument for the study of gastroesophageal reflux disease (GERD) in China, including a general information questionnaire, the Reflux Disease Questionnaire, the Quality of Life in Reflux and Dyspepsia Questionnaire, and the GERD Impact Scale. [file 1471-230X-8-37-S1.pdf]
